# Posterior Reversible Encephalopathy Syndrome Following Recreational Nitrous Oxide Use: A Case Report

**DOI:** 10.7759/cureus.106389

**Published:** 2026-04-03

**Authors:** Allyson Resnick, FNU Varnika, Patrick Patrick Leber, Bryan Dawkins, Khavir Sharieff, Mahmoud Issa

**Affiliations:** 1 Family Medicine, Nova Southeastern University Dr. Kiran C. Patel College of Osteopathic Medicine, Fort Lauderdale, USA; 2 Family Medicine, Lakeside Medical Center, Belle Glade, USA; 3 Surgery, Nova Southeastern University Dr. Kiran C. Patel College of Osteopathic Medicine, Clearwater, USA

**Keywords:** inhalation, laughing gas, nitrous oxide, posterior reversible encephalopathy, recreational

## Abstract

Posterior reversible encephalopathy syndrome (PRES) is a clinicoradiologic diagnosis characterized by acute neuropsychiatric symptoms, including headache, visual disturbances, seizures, hallucinations, and encephalopathy, in conjunction with neuroimaging findings of posterior-predominant vasogenic edema in the cerebral hemispheres. Several risk factors, including uncontrolled hypertension, eclampsia, renal failure, autoimmune diseases, and exposure to cytotoxic and immunosuppressive drugs, have been implicated in the development of PRES.

We present a rare case of PRES in a patient with a history of polysubstance abuse who presented with acute mental status changes and obtundation. The diagnosis of PRES was established based on the clinical presentation of encephalopathy and brain imaging findings demonstrating transient vasogenic edema predominantly affecting the posterior cerebral hemispheres on magnetic resonance imaging (MRI). The etiology in this case was attributed to recreational nitrous oxide misuse and intoxication, which likely resulted in vasogenic edema in the posterior lobes, leading to acute, transient neurologic dysfunction followed by recovery.

To our knowledge, the association between recreational nitrous oxide abuse and PRES has not been previously reported. This case highlights a potentially underrecognized neurological complication of nitrous oxide misuse and underscores the need for clinical vigilance and further investigation into the pathophysiologic consequences of its recreational use.

## Introduction

Posterior reversible encephalopathy syndrome (PRES) is a clinicoradiologic entity characterized by vasogenic edema, typically involving the parieto-occipital lobes. It can present with a broad range of neurological symptoms, including altered mental status, headache, seizures, visual disturbances, and encephalopathy. Known risk factors include severe hypertension, renal failure, eclampsia, autoimmune disorders, and exposure to cytotoxic and immunosuppressive drugs [1]. The proposed pathophysiology involves cerebrovascular dysregulation and/or endothelial dysfunction, leading to disruption of the blood-brain barrier and subsequent vasogenic edema [2]. The most common neuroimaging finding is vasogenic edema affecting the white matter of the occipital lobes and, less commonly, the parietal lobes [3].

Nitrous oxide (N₂O), commonly known as “laughing gas,” is widely used in clinical settings for anesthesia and analgesia. However, it is increasingly misused recreationally, typically inhaled from balloons or canisters (“whippets”). Acute excessive use has been associated with complications such as myelopathy, thromboembolism, and metabolic disturbances, including hypoxic injury due to alveolar oxygen displacement [4]. Chronic misuse leads to inactivation of vitamin B12, impairing myelin synthesis and resulting in subacute combined degeneration of the spinal cord, peripheral neuropathy, and cognitive changes [5,6].

PRES has not previously been reported as a complication of nitrous oxide abuse. This case describes an adult with a history of polysubstance abuse who presented with acute mental status changes and obtundation following recreational nitrous oxide use, leading to a clinicoradiologic diagnosis of PRES. It is likely that a combination of chronic exposure and acute intoxication acted as a precipitating factor, possibly through autonomic dysregulation, hypoxia, or endothelial injury, resulting in vasogenic edema and neurologic dysfunction. This case highlights a potentially rare but important neuropsychiatric complication of nitrous oxide abuse and underscores the need for vigilance and consideration of this exposure as a possible risk factor for PRES.

## Case presentation

A 44-year-old man with a history of polysubstance use was brought to the emergency department (ED) after being found unresponsive at home by his family. In the ED, he was unresponsive to verbal and painful stimuli, with a Glasgow Coma Scale (GCS) score of 3. On primary survey, he appeared cachectic and older than his stated age. Vital signs showed tachycardia (121 beats per minute), tachypnea (23 breaths per minute), and hypertension (162/108 mmHg). Mucous membranes were dry, consistent with significant dehydration. There was no evidence of head trauma, although multiple superficial bruises were noted on the trunk and extremities. Pupils were 4 mm and mildly reactive to direct and consensual light bilaterally. Blood glucose was 138 mg/dL (reference range: 70-110 mg/dL). The patient was intubated for airway protection, started on intravenous fluids, and a comprehensive diagnostic workup was initiated to evaluate acute metabolic encephalopathy.

**Figure 1:**
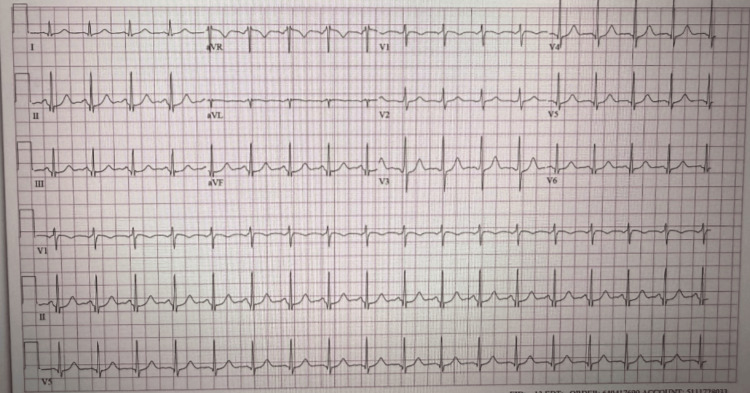
Electrocardiogram on admission showing sinus tachycardia without acute ischemic changes.

**Figure 2:**
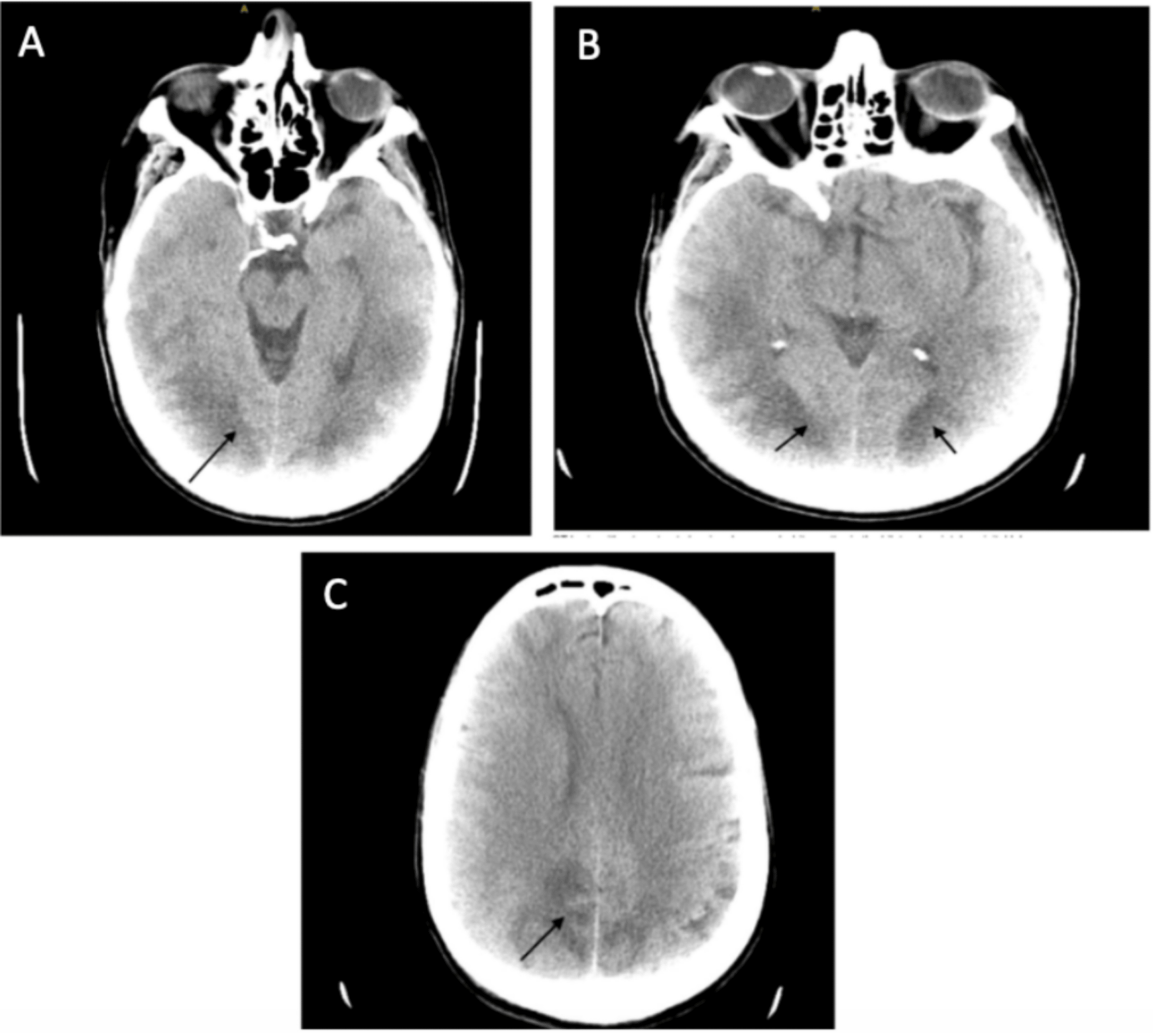
Non-contrast computed tomography of the brain. Axial non-contrast computed tomography images demonstrate decreased white matter attenuation in the parieto-occipital lobes bilaterally (arrows). (A) Axial slice at the basal ganglia level showing early posterior hypodensity. (B) Axial slice at the ventricular level demonstrating bilateral parieto-occipital white matter changes. (C) Superior axial slice confirming persistent posterior white matter hypodensity.

**Figure 3:**
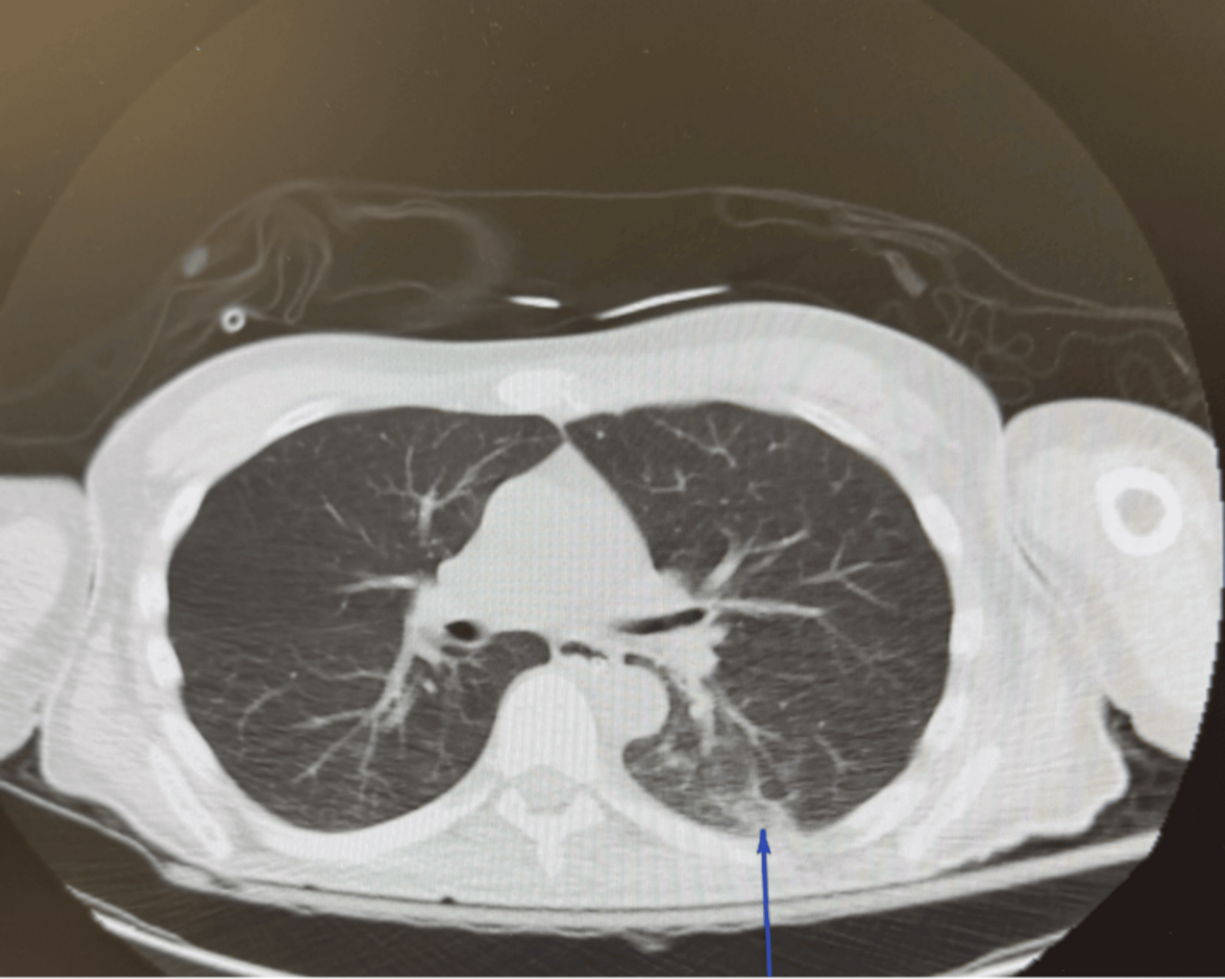
Computed tomography of the chest showing patchy consolidation in the posterior left lower pulmonary lobe (blue arrow).

Electrocardiography showed sinus tachycardia without evidence of acute ischemia or injury (Figure 1). Toxicology screening was positive for benzodiazepines and cannabis. The metabolic panel (Table 1) demonstrated pre-renal azotemia, with a blood urea nitrogen (BUN)/creatinine ratio >20:1 (reference ranges: BUN, 7-18 mg/dL; creatinine, 0.50-1.20 mg/dL), hypernatremia (157 mg/dL; reference range: 136-145 mg/dL), and elevated lactate (4.9 mmol/L; reference range: 0.4-1.9 mmol/L), suggesting possible prolonged downtime and severe dehydration. Non-contrast computed tomography (CT) of the brain revealed symmetric hypodensities in the subcortical white matter of both cerebral hemispheres, raising concern for toxic metabolic encephalopathy or PRES (Figures 2A-2C). Chest CT showed patchy consolidations in the posterior segment of the left lower lobe (Figure 3). The patient was admitted to the intensive care unit (ICU). Hemodynamic status was optimized, seizure prophylaxis was initiated, and further evaluation was undertaken to determine the underlying cause of PRES in this patient.

**Table 1:** Laboratory data on admission.

Variable	Reference range (hospital)	On current presentation
White blood cell count	4.8-10.8 × 10³/µL	9.6
Red blood cell count	4.15-4.89 × 10⁶/µL	3.41
Sodium	136-145 mg/dL	157
Blood urea nitrogen	7-18 mg/dL	57
Creatinine	0.50-1.20 mg/dL	1.65
Alanine aminotransferase	12-78 U/L	115
Aspartate aminotransferase	15-37 U/L	598
Thyroid-stimulating hormone	0.358-0.3740 uIU/mL	0.101
T3	2.18-3.98 pg/mL	1.45
Acetaminophen	10.0-30.0 ug/mL	<1.0
Salicylate	2.8-20.0 mg/dL	<0.2
Ethanol	0-10 mg/dL	<3.0
Vitamin B12	232-1245 pg/mL	303
PCO_2_, art	36-44 mmHG	46.3
PO_2_, art	90-100 mmHg	400
pH	7.38-7.42	7.37
Lactic acid	0.4-1.9 mmol/L	4.9
Glucose	70-110 mg/dL	138

**Figure 4:**
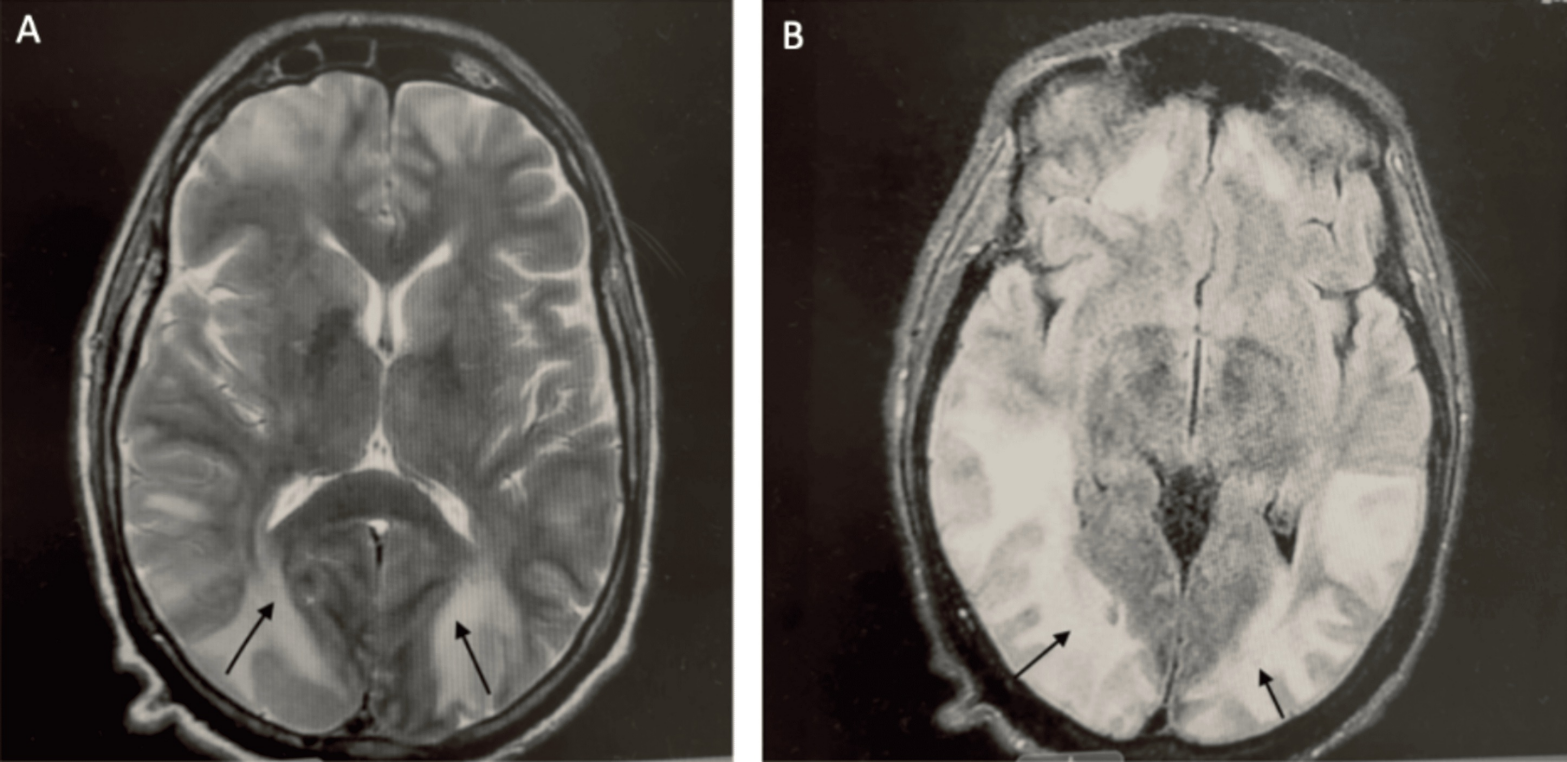
Axial fluid-attenuated inversion recovery (FLAIR) and T2-weighted MRI sequences of the brain showing posterior abnormalities. Axial fluid-attenuated inversion recovery and T2-weighted MRI sequences demonstrate bilateral subcortical and cortical hyperintensities in the parieto-occipital regions (arrows). (A) Axial FLAIR image showing symmetric posterior hyperintensities. (B) Axial T2-weighted image confirming bilateral parieto-occipital involvement.

After 24 hours in the ICU, renal function improved with intravenous fluid resuscitation; however, neurologic assessment revealed persistent deficits, including rightward gaze deviation and decerebrate posturing. Magnetic resonance imaging (MRI) of the brain demonstrated diffuse vasogenic edema involving the bilateral parieto-occipital regions, with relative sparing of the basal ganglia and brainstem, consistent with PRES (Figures 4A, 4B). Additionally, diffuse cortical petechial microhemorrhages were noted, without midline shift or basilar cisternal effacement. Video electroencephalography (EEG) showed no evidence of epileptic activity.

**Figure 5:**
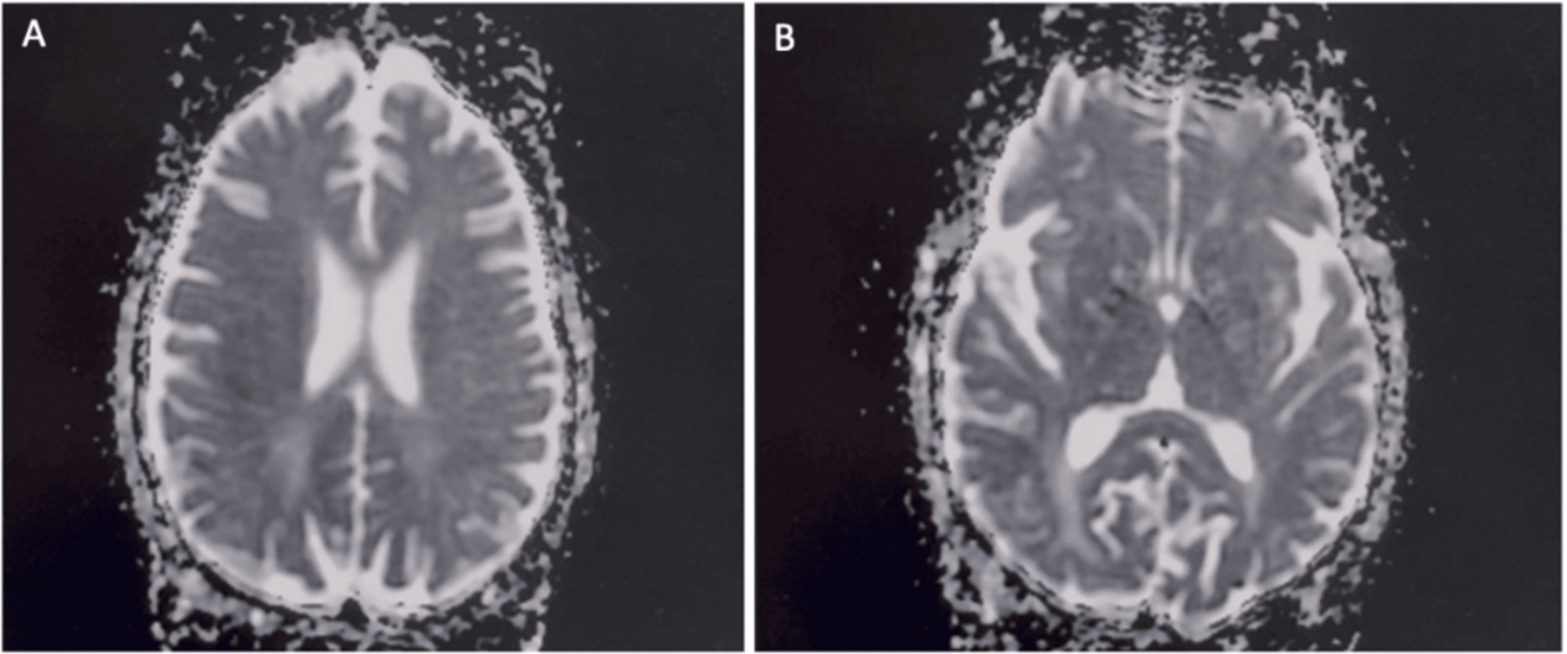
Follow-up brain MRI demonstrating interval improvement. Follow-up MRI of the brain demonstrates marked improvement of previously noted signal abnormalities within the supratentorial compartment, with minimal residual gliosis in the posterior cortices. (A) Axial image showing near-resolution of prior posterior hyperintensities. (B) Axial image confirming significant interval improvement with minimal residual signal abnormality.

Encephalopathy secondary to PRES was managed with supportive care, including intravenous fluids and electrolyte replacement, resulting in successful extubation. Neurologic function gradually improved with rehabilitation, and the patient was discharged on hospital day 31. A follow-up MRI performed three weeks later showed marked improvement in the previously noted signal abnormalities within the ventral and posterior supratentorial compartments, with minimal residual gliosis and abnormal signal in the right greater than left posterior cortices (Figures 5A, 5B).

**Figure 6:**
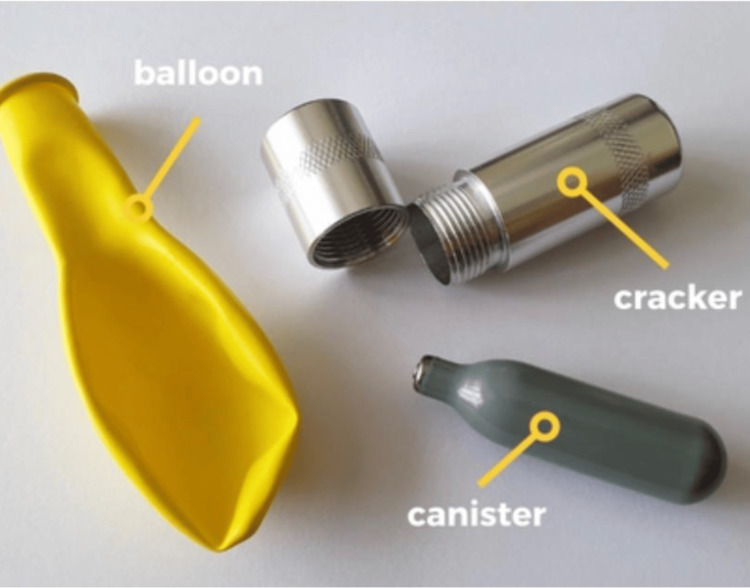
Representative equipment commonly used for recreational nitrous oxide (N₂O) misuse. Source: [7] (Creative Commons Attribution 2.0 Generic (CC BY 2.0)).

The patient lived with his father, who had severe Alzheimer’s dementia. He had a longstanding history of polysubstance abuse, with multiple prior evaluations for opioid and benzodiazepine overdose, as well as a burn injury related to Freon use a few weeks before this admission. Additional history obtained from family members revealed that, in addition to narcotics, the patient routinely abused nitrous oxide recreationally. Numerous deflated rubber balloons (“whippets”) and canisters (Figure 6), suspected to have been used for nitrous oxide inhalation, were found in his home. Small canisters typically contain 8 g, whereas larger canisters may contain up to 600 g.

In retrospect, given the number of deflated balloons found at his residence, although the total amount of inhaled nitrous oxide is difficult to quantify, it was likely substantial, raising suspicion for acute intoxication superimposed on chronic use.

## Discussion

Based on our literature review, this appears to be the first reported case of PRES associated with nitrous oxide use. This case highlights a rare neurological complication related to nitrous oxide misuse. Although myelopathy with subacute combined degeneration of the spinal cord and peripheral neuropathy are the most commonly documented adverse outcomes of chronic nitrous oxide exposure [5,6], our patient developed PRES, an acute and potentially reversible disorder of cerebrovascular regulation. In a case series of 119 patients using nitrous oxide, 98 demonstrated MRI findings of myeloneuropathy consistent with subacute combined degeneration, and none had findings suggestive of PRES [6].

​​The diagnosis of PRES in the setting of polysubstance use and comorbid conditions can be challenging. In cases of metabolic encephalopathy, a combination of clinical assessment and timely diagnostic evaluation is essential to identify PRES and initiate supportive management to mitigate neurologic dysfunction. A brain CT demonstrating symmetric subcortical hypodensities may raise concern for toxic metabolic encephalopathy or PRES. MRI, including initial or serial imaging, can confirm the diagnosis by demonstrating characteristic parieto-occipital vasogenic edema. When the etiology remains unclear, additional history from family members or close contacts may be informative. In this case, the patient’s brother reported finding numerous deflated balloons and canisters, which helped implicate nitrous oxide as a potential etiologic factor. A standard nitrous oxide canister typically contains approximately 8 g of gas, equivalent to about 4 L of nitrous oxide [6].

Recreational use of nitrous oxide is increasing due to its euphoric effects. However, adverse effects such as nitrous oxide-induced myeloneuropathy, subacute combined degeneration of the spinal cord, peripheral neuropathy, and functional vitamin B12 deficiency can lead to significant neuropsychiatric consequences. High-dose or prolonged use, particularly over a short period, appears to increase the risk of complications. Even the use of a small number of canisters may produce symptoms. In addition, larger cylinders used for recreational purposes can cause frostbite injuries due to direct exposure to liquefied gas [6]. Notably, our patient had a history of second-degree skin burns three weeks prior to admission, likely related to canister use.

While PRES has been associated with other recreational drugs, particularly cocaine [3], this case suggests nitrous oxide as a potential precipitating factor, thereby expanding the spectrum of inhalant-related brain injury. Although the exact mechanism by which nitrous oxide may contribute to PRES remains unclear, proposed mechanisms include transient hypertension or autonomic instability during intoxication, cerebral hypoxia due to oxygen displacement, and direct endothelial injury. These processes may disrupt the blood-brain barrier and impair cerebrovascular autoregulation, ultimately resulting in posterior-predominant vasogenic edema [8].

This case underscores the importance of considering nitrous oxide use in the differential diagnosis of PRES, particularly in patients who lack common risk factors such as chronic hypertension, renal disease, or exposure to immunosuppressive therapy. Although this patient presented with elevated blood pressure, prior emergency department visits documented normal readings (116/69 mmHg), arguing against chronic hypertension. In addition, toxicology screening was negative for cocaine, renal function was preserved (glomerular filtration rate 71 mL/min/1.73 m²), and there was no history of immunosuppressive therapy.

PRES is potentially reversible with early supportive management; however, delayed diagnosis may result in permanent neurologic deficits or death. Therefore, timely recognition of this association is critical [1]. From a public health perspective, the increasing recreational use of nitrous oxide, driven in part by the widespread availability of canisters and balloons [4], highlights the need for heightened awareness and careful evaluation of its complications. This case suggests that the risks of nitrous oxide misuse should be expanded to include acute, potentially life-threatening cerebrovascular syndromes such as PRES, particularly in patients presenting with encephalopathy and abnormal neuroimaging findings.

## Conclusions

This case highlights PRES in the context of suspected recreational misuse of nitrous oxide canisters or balloons (“whippets”). Nitrous oxide inhalant abuse should be considered a potential cause of PRES, particularly in patients presenting with encephalopathy and atypical neurologic findings, along with imaging evidence of vasogenic edema involving the occipital and parietal lobes. Prompt recognition of PRES allows for identification of the precipitating factor and initiation of supportive care to mitigate neurologic injury and optimize outcomes. PRES has a broad differential diagnosis and is associated with several conditions, including severe hypertension, renal dysfunction, autoimmune diseases, exposure to cytotoxic or immunosuppressive medications, and metabolic disturbances. In this patient, these alternative etiologies were carefully evaluated during the diagnostic workup. The temporal association with nitrous oxide misuse, together with the clinical presentation and characteristic neuroimaging findings, supports nitrous oxide exposure as the most likely contributing factor in this case.
